# ﻿A new bamboo-feeding species of the genus *Pseudosymplanella* Che, Zhang & Webb, 2009 (Hemiptera, Caliscelidae, Ommatidiotinae) from China

**DOI:** 10.3897/zookeys.1186.111838

**Published:** 2023-12-11

**Authors:** Nian Gong, Xiang-Sheng Chen, Lin Yang

**Affiliations:** 1 Guizhou Provincial Engineering Research Center of Medical Resourceful Healthcare Products, Guiyang Healthcare Vocational University, Guiyang, Guizhou, 550081, China Guiyang Healthcare Vocational University Guiyang China; 2 Institute of Entomology, Guizhou University, Guiyang, Guizhou, 550025, China Guizhou University Guiyang China

**Keywords:** Augilini, bamboo, identification key, morphology, Oriental region, plant­hopper, taxonomy

## Abstract

A new planthopper species, *Pseudosymplanellamaxima***sp. nov.**, belonging to the genus *Pseudosymplanella* (Hemiptera: Fulgoromorpha: Caliscelidae: Augilini), is described and illustrated, from China. In common with other Chinese Augilini, the new species feeds exclusively on bamboo. Additionally, a key to the two species of *Pseudosymplanella* is provided.

## ﻿Introduction

The family Caliscelidae Amyot & Audinet-Serville, 1843, a worldwide distributed group, is divided into two subfamilies: Caliscelinae (including tribes Caliscelini and Peltonotellini) and Ommatidiotinae (including tribes Ommatidiotini, Augilini and Adenissini) (Gnezdilov and Wilson 2006; Emeljanov 2008; Gnezdilov 2008). The two subfamilies can be separated by nymphal characters (Gnezdilov and Wilson 2006), disparities in the first metatarsomere and the degree of aedeagus reduction (Gnezdilov and Bourgoin 2009).

Modern fauna of the tribe Augilini Baker, 1915 contains 16 genera and 41 species, including the new species described below (Zhang et al. 2020; Gong et al. 2021; Bourgoin 2023). A fossil genus and species was documented in Dominican amber in the New World (Bourgoin et al. 2015b). Now, seven genera and 21 species are recorded from southern China. It is worth noting that all these species have been documented to be bamboo-feeders (Chen et al. 2014; Gong et al. 2018, 2020, 2021; Zhang et al. 2020).

The planthopper genus *Pseudosymplanella* was established by Che, Zhang and Webb (2009) based on a single species, *Pseudosymplanellanigrifasciata*, from China and Thailand, and placed in the tribe Augilini of the subfamily Ommatidiotinae (Hemiptera: Fulgoroidea: Caliscelidae).

In the present paper, a new species, *Pseudosymplanellamaxima* sp. nov. is described from Yunnan Province, China. Descriptions and illustrations are given, generic characteristics are redefined, and a key to species of *Pseudosymplanella* is provided.

## ﻿Material and methods

Terminology used for the external morphology and the male genitalia mainly follows the classifications proposed by Fennah (1987) and Chan and Yang (1994). The standard terminology for hind and forewing venation adheres to the principle outlined by Bourgoin et al. (2015a). The methodology for describing the description of the female genitalia mainly follows Bourgoin’s (1993) approach. Dry specimens were used for the descriptions and illustrations. External morphology was observed under a stereoscopic microscope and characters were measured with an ocular micrometer. All dimensions were expressed in millimeters (mm); the body length was measured from the apex of the head to the apex of the forewing in repose. The genital segments of the examined specimens were subjected to maceration in a 10% NaOH and subsequently observed in glycerin jelly for illustration using a Leica MZ 12.5 stereomicroscope. Photographs were captured with a KEYENCE VHX-1000 system. Illustrations were scanned with CanoScan LiDE 200 and imported into Adobe Photoshop CS6 for labeling and plate composition. The dissected male genitalia were preserved in glycerine and then stored in small plastic tubes, which were pinned together with the specimens.

The type specimens and materials examined were deposited in the Institute of Entomology, Guizhou University, Guiyang, China (**IEGU**).

## ﻿Taxonomy

### ﻿Order Hemiptera Linnaeus, 1758

**Suborder Fulgoromorpha Evans, 1946**

**Family Caliscelidae Amyot & Audinet-Serville, 1843**

**Ommatidiotinae Fieber, 1875**

**Tribe Augilini Baker, 1915**

#### ﻿ Pseudosymplanella

Taxon classificationAnimaliaHemipteraCaliscelidae

Che, Zhang & Webb, 2009

EB8E79CE-2F9D-5056-8879-35D49A037392

Figs 1–4 , 5–15 , 16–21

Pseudosymplanella Che, Zhang & Webb, 2009: 49.

##### Type species.

*Pseudosymplanellanigrifasciata* Che, Zhang & Webb, 2009, by original designation.

**Figures 1–4.**
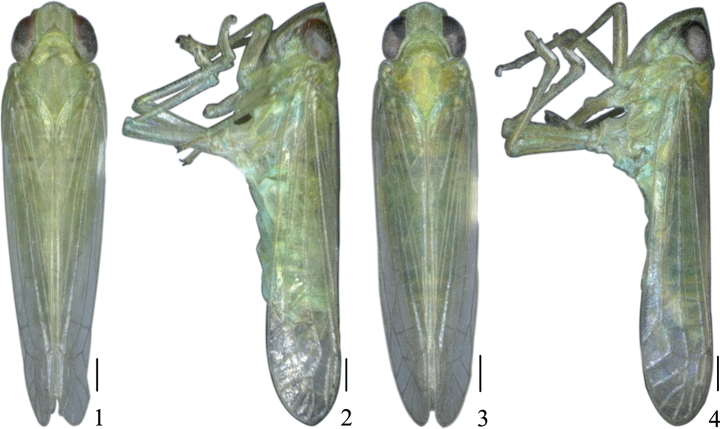
*Pseudosymplanellamaxima* Gong, Yang & Chen, sp. nov. **1** male habitus, dorsal view **2** male habitus, lateral view **3** female habitus, dorsal view **4** female habitus, lateral view. Scale bars: 0.5 mm (**1–4**).

##### Diagnosis.

Head with eyes as wide as or slightly narrower than pronotum; vertex with anterior margin a little convex or straight; second segment of antenna with a black transverse spot near apex. Mesonotum sometimes with pit along lateral margin, maximum width wider than medial length. Male with pygofer in lateral view, dorsal margin shorter than ventral margin, posterior margin with a rather slender and long process; genital style in lateral view elongate or rather broad; aedeagus simple, tubular, slightly ventrally curved. Female genitalia with gonoplacs rounded or triangular in lateral view.

**Figures 5–15.**
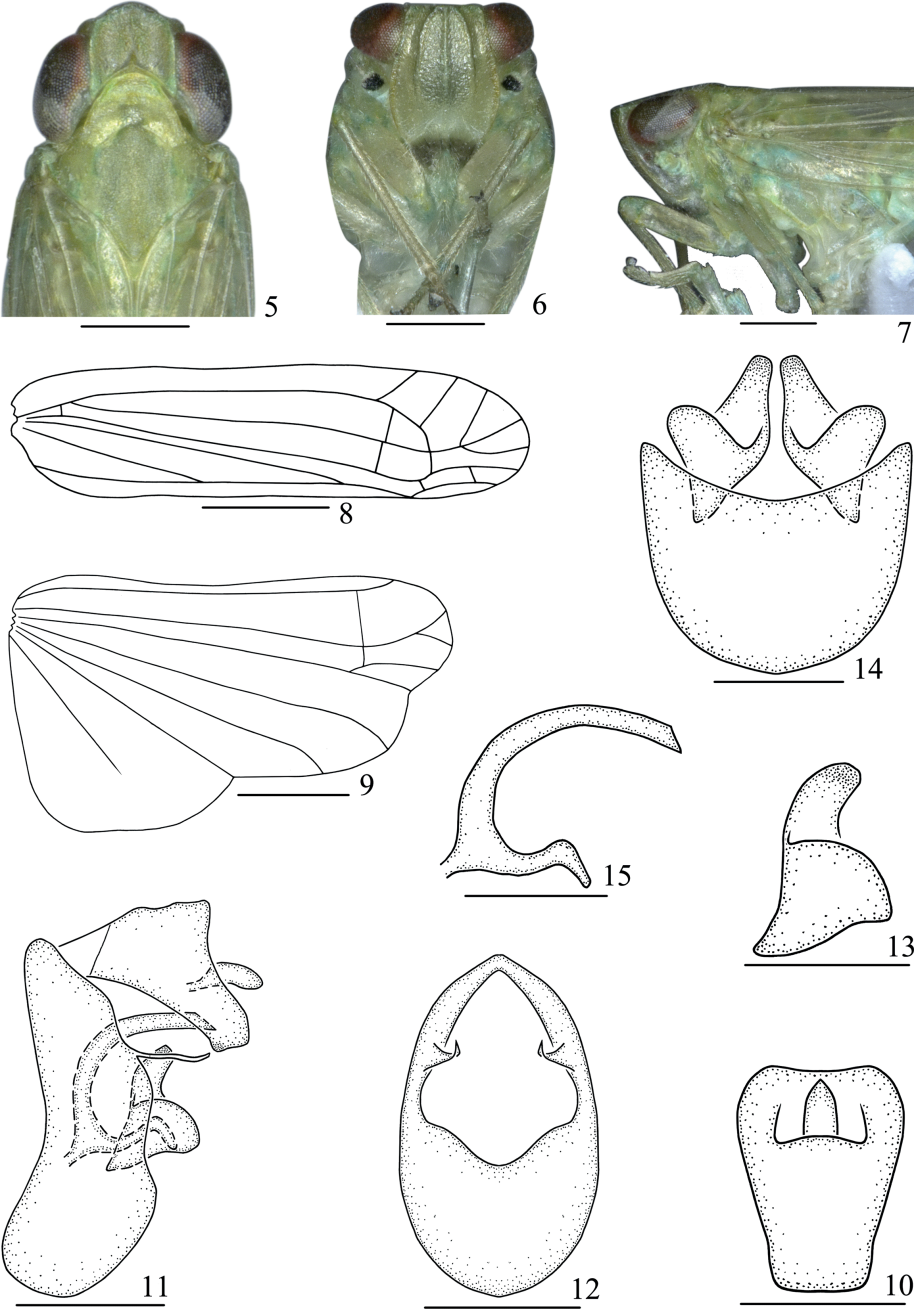
*Pseudosymplanellamaxima* Gong, Yang & Chen, sp. nov., male **5** head and thorax, dorsal view **6** face **7** head and thorax, lateral view **8** forewing **9** hindwing **10** anal segment, dorsal view **11** male genitalia, lateral view **12** pygofer, posterior view **13** genital styles, lateral view **14** pygofer and genital styles, ventral view **15** aedeagus, lateral view. Scale bars: 1 mm (**8, 9**); 0.5 mm (**5–7**); 0.3 mm (**11**); 0.2 mm (**10, 12–15**).

**Figures 16–21.**
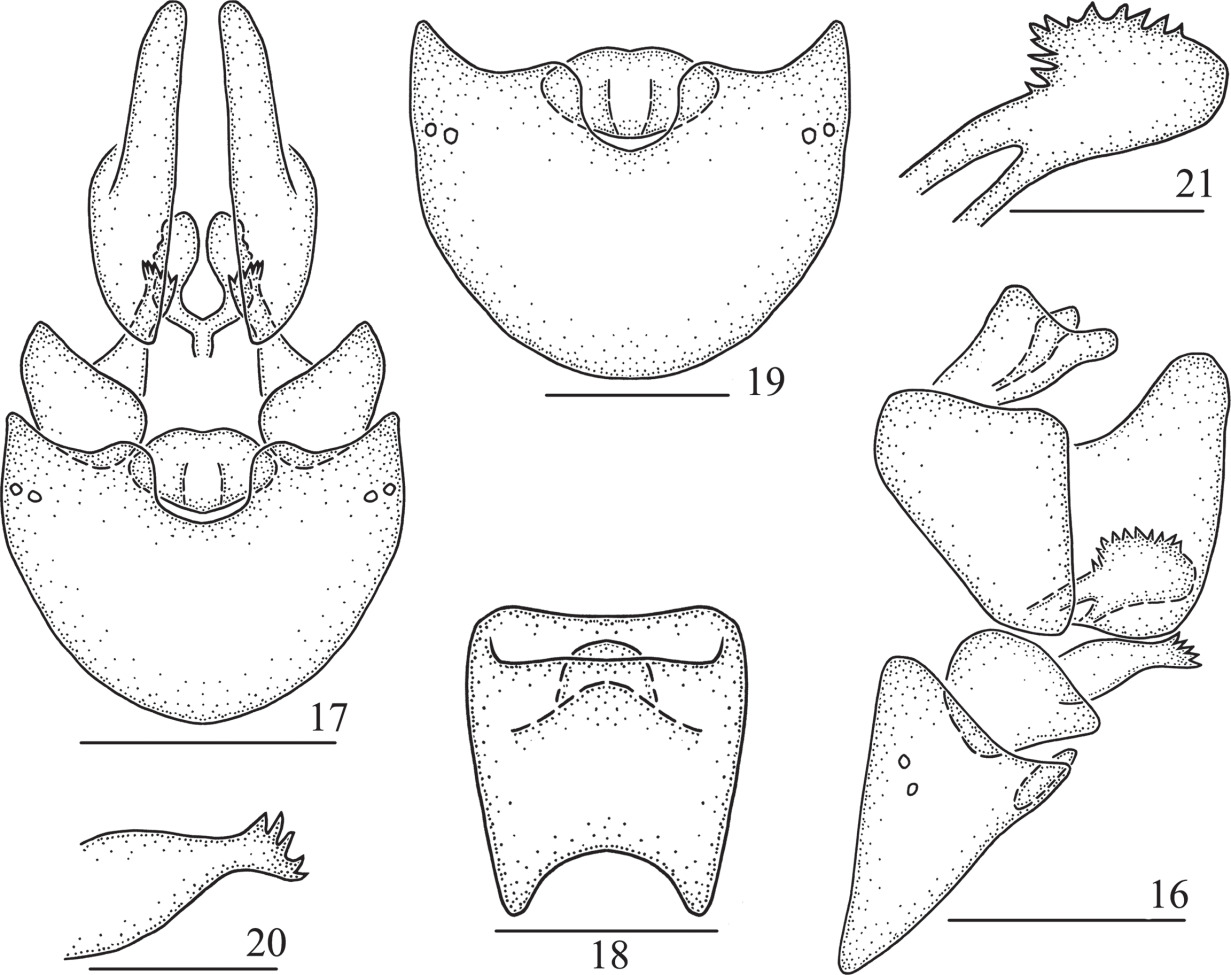
*Pseudosymplanellamaxima* Gong, Yang & Chen, sp. nov., female **16** genitalia, lateral view **17** genitalia, ventral view **18** anal segment, dorsal view **19** abdominal sternite VII, ventral view **20** gonapophysis VIII, lateral view **21** gonapophysis IX, lateral view. Scale bars: 0.5 mm (**16, 17**); 0.3 mm (**19**); 0.2 mm (**18, 20, 21**).

##### Host plant.

Bamboo.

##### Distribution.

**Figure 22.**
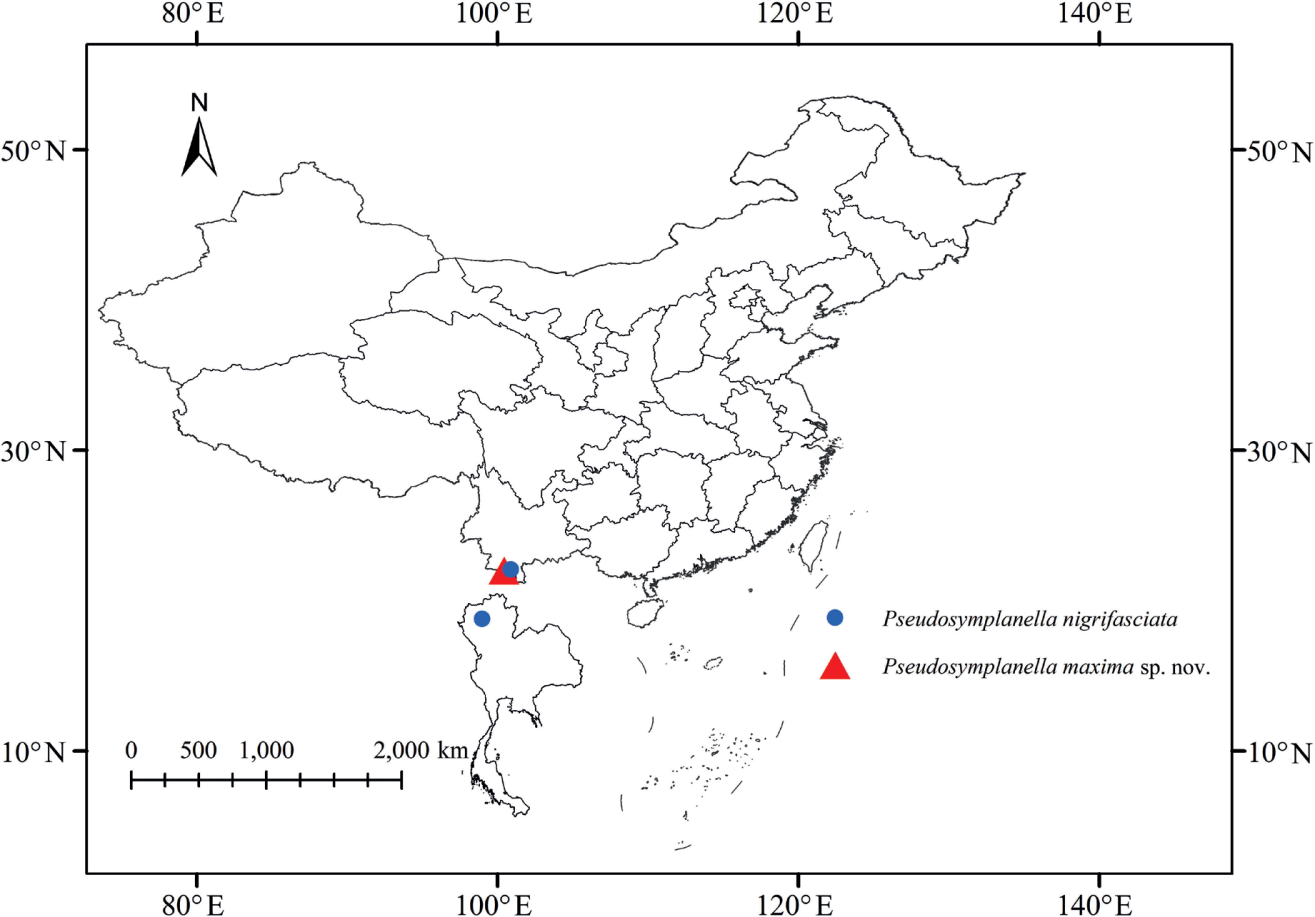
Geographic distributions of species of *Pseudosymplanella* Che, Zhang & Webb, 2009.

Southern China (Yunnan Province) and Thailand (Fig. 22).

### ﻿Key to species of genus *Pseudosymplanella* Che, Zhang & Webb, 2009

**Table:** 

1	Body mainly brown, anal segment with anal pore located at mid-length, posterior margin of pygofer in profile with thick and short process near dorsal margin	***P.nigrifasciata***
–	Body grass green, anal segment with anal pore located in apical half, posterior margin of pygofer in profile with slender and long process near mid-length	***P.maxima* sp. nov.**

#### ﻿ Pseudosymplanella maxima sp. nov.

Taxon classificationAnimaliaHemipteraCaliscelidae

4BF4B3BC-BD75-5B49-BC5B-72A0DFEE2D74

https://zoobank.org/A2F51374-787E-4703-8C71-694657D3A9B2

Figs 1–4 , 5–15 , 16–21

##### Description.

***Measurements*.** Body length including forewing: male 5.1 mm (*N* = 1), female 6.0–6.2 mm (*N* = 3); forewing length: male 4.2 mm (*N* = 1), female 5.0–5.2 mm (*N* = 3).

***Coloration*.** Body (Figs 1–4) grass green. Eyes reddish brown, ocelli orange red. Second segment of antenna (Fig. 6) with a black transverse spot near apex. Clypeus (Fig. 6) with basal half brown.

***Head and thorax*.** Head (Fig. 5) with eyes as wide as pronotum. Vertex (Fig. 5) with length in middle line 0.8 times than width at base. Frons (Fig. 6) with length in middle line 1.1 times than maximum width. Pronotum (Fig. 5) with length in middle line shorter than vertex (0.8:1). Mesonotum (Fig. 5) 1.2 times as long as vertex and pronotum together in middle line. Forewing (Fig. 8) longer in middle line than broad at widest part (3.8:1); veins distinct, without nodal line, R and MP with common stem; ScP, R and CuA single, MP with three branches, Pcu uniting A1 at basal half of clavus. Hindwing (Fig. 9) with length 1.7 times as long as broad at widest part, ScP and RP single, MP and CuA with two branches. Legs relatively long, hind tibia with a single lateral tooth; spinal formula of hind leg 6-0-0.

***Male genitalia*.** Anal segment (Fig. 10) in dorsal view with length 1.3 times longer in mid-line than widest part, apical margin slightly concave; anal pore located at apical half; in lateral view (Fig. 11) dorsal margin sinuated, ventral margin slightly concave near apex with a small process, broadening distally and abruptly narrowed subapically. Pygofer in lateral view (Fig. 11) with dorsal margin distinctly shorter than ventral margin, posterior margin sinuated with a rather slender and long process near mid-length; in posterior view (Fig. 12), nearly oval, with length 1.7 times longer in mid-line than widest part; in ventral view (Fig. 14), posterior margin broadly concave. Genital style in lateral view (Fig. 13) rather broad, nearly triangle, apical margin roundly convex; a strong finger-like process apically arising from dorsal margin, slightly curved. Aedeagus (Fig. 15) simple, tubular, slightly ventrally curved.

***Female genitalia*.** Anal segment small, short, in dorsal view (Fig. 18) nearly quadrangle, anal pore near apex. Abdominal sternite VII in ventral view (Fig. 19) rather large and broad, behind the posterior margin with a small oval ossification flake. Gonapophysis VIII (first valvula) (Fig. 20) elongate, with five spines at apical margin. Gonapophysis IX (second valvula) (Fig. 21) with two symmetrical lobes, each lobe with many spines at dorsal margin. Gonoplac (third valvula) (Fig. 16) triangular, apical margin rounded.

##### Host plant.

Bamboo.

##### Distribution.

Southwestern China (Yunnan Province) (Fig. 22).

##### Type material.

***Holotype***: ♂, China: Yunnan Province, Menghai County, Mengzhe Reservoir (22°08'N, 100°26'E), 2019-X-4, Nian Gong. ***Paratypes***: 1♂3♀, data same as holotype.

##### Etymology.

The specific name is derived from the Latin word “maximus”, referring to the long process of the pygofer.

##### Remarks.

This new species is closely related to *P.nigrifasciata* Che, Zhang & Webb, 2009, but differs in: 1) body mainly green, without stripe (body brown, with stripe in *P.nigrifasciata*); 2) anal segment with anal pore located in apical half (anal pore located at mid-length in *P.nigrifasciata*); and 3) posterior margin of pygofer in profile with slender and long process near the mid-length (posterior margin of pygofer in profile with thick and short process near dorsal margin in *P.nigrifasciata*).

## Supplementary Material

XML Treatment for Pseudosymplanella

XML Treatment for Pseudosymplanella maxima

## ﻿Additional information

### Author ORCIDs

Nian Gong https://orcid.org/0000-0002-8878-5337

Xiang-Sheng Chen https://orcid.org/0000-0001-9801-0343

Lin Yang https://orcid.org/0000-0002-7841-5156

### Data availability

All of the data that support the findings of this study are available in the main text.
